# Correction: Berghäll et al. The Evolution of Blood Cell Phenotypes, Intracellular and Plasma Cytokines and Morphological Changes in Critically Ill COVID-19 Patients. *Biomedicines* 2022, *10*, 934

**DOI:** 10.3390/biomedicines11112965

**Published:** 2023-11-03

**Authors:** Elisabeth Berghäll, Michael Hultström, Robert Frithiof, Miklos Lipcsey, Victoria Hahn-Strömberg

**Affiliations:** 1 Department of Surgical Sciences, Anesthesiology and Intensive Care, Uppsala University, 75185 Uppsala, Sweden; michael.hultstrom@mcb.uu.se (M.H.); robert.frithiof@surgsci.uu.se (R.F.); miklos.lipcsey@surgsci.uu.se (M.L.); 2 Department of Medical Cell Biology, Uppsala University, 75123 Uppsala, Sweden; victoria.stromberg@mcb.uu.se; 3 Hedenstierna Laboratory, CIRRUS, Anesthesiology and Intensive Care, Department of Surgical Sciences, Uppsala University, 75185 Uppsala, Sweden

## 1. Error in Table

In original publication [1] there was a mistake in Table 1. The rows with the Mortality of 30 day and 90 day respectively should switch columns so that 10 (13) and 15 (19) patients died in the All Patients group, and 3 (12) and 4 (16) patients died in the Flow Cytometry Patient group. The corrected Table 1 appears below.

## 2. Text Correction

We hereby state that there was an error in the original publication and a mistake was made in the text as below. 

In the abstract, subsection Results, fifth row:

“The expression levels of intracellular tumor necrosis factor alpha (TNFα) and IL-1 receptor type 2 in leukocytes were higher (*p* < 0.001) as well as plasma levels of TNFα, IL-2, IL-6, IL-8, IL-10 (*p* < 0.001), interferon gamma (IFNγ) (*p* < 0.01), and granulocyte-macrophage colony-stimulating factor (GM-CSF) (*p* < 0.05)”.

The word higher in the sentence above should be lower. And then the whole sentence needs to be revised, suggestion below: 

“The expression levels of intracellular tumor necrosis factor alpha (TNFα) and IL-1 receptor type 2 in leukocytes were lower (*p* < 0.001), and the plasma levels of TNFα, IL-2, IL-6, IL-8, IL-10 (*p* < 0.001), interferon gamma (IFNγ) (*p* < 0.01), and granulocyte-macrophage colony-stimulating factor (GM-CSF) (*p* < 0.05) were higher in patients with severe COVID-19 than in the control group”.

The authors state that the scientific conclusions are unaffected. This correction was approved by the Academic Editor. The original publication has also been updated.

## Figures and Tables

**Table 1:** Demographic and clinical characteristics.

	All Patients	Flow Cytometry (FCM) Patients
	*n* = 78	*n* = 25
Age (years: mean ± SD)	61 ± 13	60 ± 13
Weight (kg: mean ± SD)	90 ± 24	92 ± 26
BMI (kg/m^2^: mean ± SD)	30 ± 7	31 ± 8
Female (*n*, %)	20 (38)	6 (24)
SAPS3 (mean ± SD)	53 ± 10	53 ± 9
LPC (10^9^/L: mean ± SD)		8.5 ± 4.1
Comorbidities		
Chronic pulmonary disease (*n*, %)	21 (27)	6 (24)
Hypertension (*n*, %)	37 (47)	11 (44)
Heart failure (*n*, %)	4 (5)	2 (8)
Ischemic heart disease (*n*, %)	7 (9)	3 (12)
Previous thromboembolic event (*n*, %)	7 (9)	2 (8)
Malignancy (*n*, %)	6 (8)	2 (8)
Diabetes mellitus (*n*, %)	19 (24)	4 (16)
Neurologic disease (*n*, %)	3 (4)	3 (12)
Non-smoker (*n*, %)	59 (77)	18 (72)
Medications prior to admission		
Steroid treatment (*n*, %)	7 (9) ^1^	5 (20) ^1^
ACEi or ARB treatment (*n*, %)	23 (30)	9 (36)
Anticoagulant treatment (*n*, %)	17 (22)	4 (16)
Mortality and organ failure in the ICU		
Mortality of 30 days (*n*, %)	10 (13)	3 (12)
Mortality of 90 days (*n*, %)	15 (19)	4 (16)
PaO_2_/FiO_2_ ratio of <13.3 kPa (*n*, %)	44 (82) ^2^	16 (89) ^2^
CRRT (*n*, %)	10 (13)	9 (36)
ICUAW (*n*, %)	10 (13)	8 (32)
Medication in the ICU		
Steroid treatment in the ICU (*n*, %)	13 (18)	6 (29)

^1^ missing values in 5 and 4 of the patients, respectively; ^2^ missing values in 34 and 8 of the patients. Abbreviations: Body Mass Index (BMI), SAPS3, Simplified Acute Physiology Score 3; LPC, leukocyte particle concentration; ICU, intensive care unit; ACEi, angiotensin-converting enzyme inhibitor; ARB, angiotensin receptor blocker; PaO_2_/FiO_2_ ratio, arterial oxygen partial pressure-to-fractional inspired oxygen ratio; CRRT, continuous renal replacement therapy; ICUAW, ICU-acquired weakness.
