# Supplementary material for: The Evolution of Blood Cell Phenotypes, Intracellular and Plasma Cytokines and Morphological Changes in Critically Ill COVID-19 Patients
Source: Biomedicines. 2022 Apr 19;10(5):934. doi: 10.3390/biomedicines10050934 (PMC9138896; doi:10.3390/biomedicines10050934)
Supplement: Supplementary file 1 [file biomedicines-10-00934-s001.zip › Figure S2.pdf]

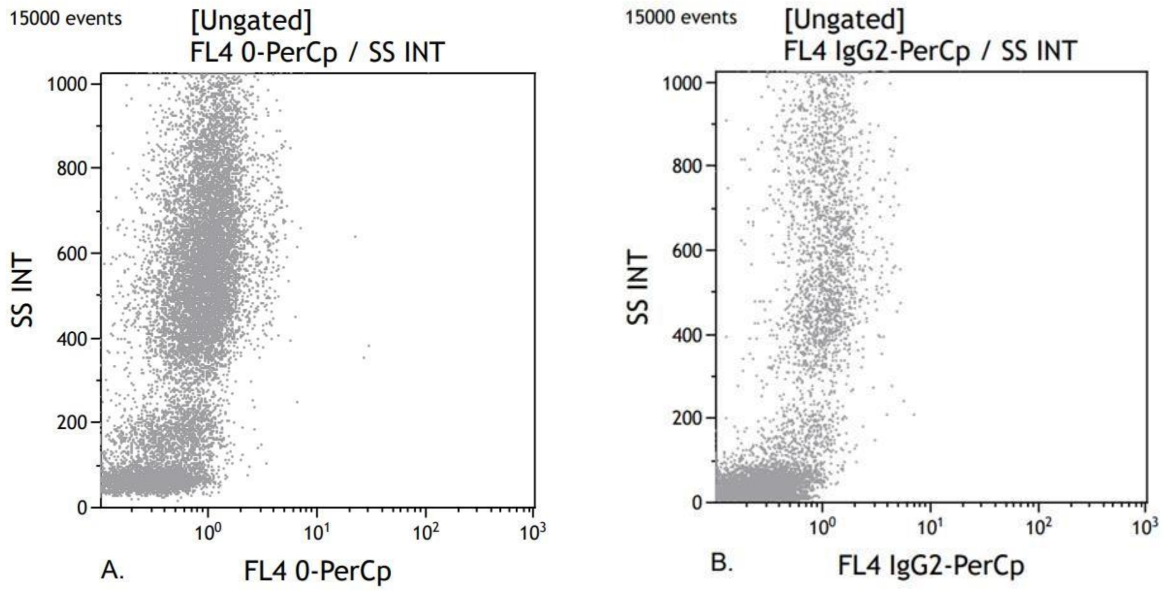

Figure S2. Negative controls for Flowcytometry (FCM) analysis. (**A**) FCM scatterplot of an unstained sample. (**B**) FCM scatterplot for the isotypic control of IgG2.
