# Supplementary material for: The Evolution of Blood Cell Phenotypes, Intracellular and Plasma Cytokines and Morphological Changes in Critically Ill COVID-19 Patients
Source: Biomedicines. 2022 Apr 19;10(5):934. doi: 10.3390/biomedicines10050934 (PMC9138896; doi:10.3390/biomedicines10050934)
Supplement: Supplementary file 1 [file biomedicines-10-00934-s001.zip › Table S1.pdf]

Table S1. Flow cytometry markers.

Antibody	Fluorochrome	Clone	Isotype
CD8	FITC	BW135/80	IgG2gk
CD33	FITC	AC104.3E3	IgG1λ
CD45	FITC	5B1	IgG2aκ
CD59	FITC	REA496	IgG1
CD127	FITC	MB15-18C9	IgG2aκ
IL-6	FITC	REA1037	IgG1
IL-8	FITC	REA731	IgG1
CD4	PE	M-T466	IgG1κ
CD25	PE	4E3	IgG2bκ
IL-10	PE	REA842	IgG1
HLA DR	PE	AC122	IgG2aκ
CD14	PerCP	TÜK4	IgG2aκ
CD45	PerCP	5B1	IgG2ak
CD2	APC	LT1	IgG2aκ
CD19	APC	REA675	IgG1
CD55	APC	JS11	IgG1k
CD158d	APC	REA768	IgG1λ
CD203c	APC	FR3-16A11	IgG1κ
IL1R2	APC	REA689	IgG1
TNFα	APC	REA656	IgG1

Abbreviations: Human Leukocyte Antigen (HLA), Interleukin (IL), Interleukin 1 receptor 2 (IL1R2), Tumor Necrosis Factor alfa (TNFα), Fluoroescein Isothiocyanate (FITC), Phycoerythrin (PE), Peridinin-chlorophyllprotein Complex (PerCP), Allophycocyanin Conjugate (APC).
