# Supplementary figures and images for: The Evolution of Blood Cell Phenotypes, Intracellular and Plasma Cytokines and Morphological Changes in Critically Ill COVID-19 Patients
Source: Biomedicines. 2022 Apr 19;10(5):934. doi: 10.3390/biomedicines10050934 (PMC9138896; doi:10.3390/biomedicines10050934)

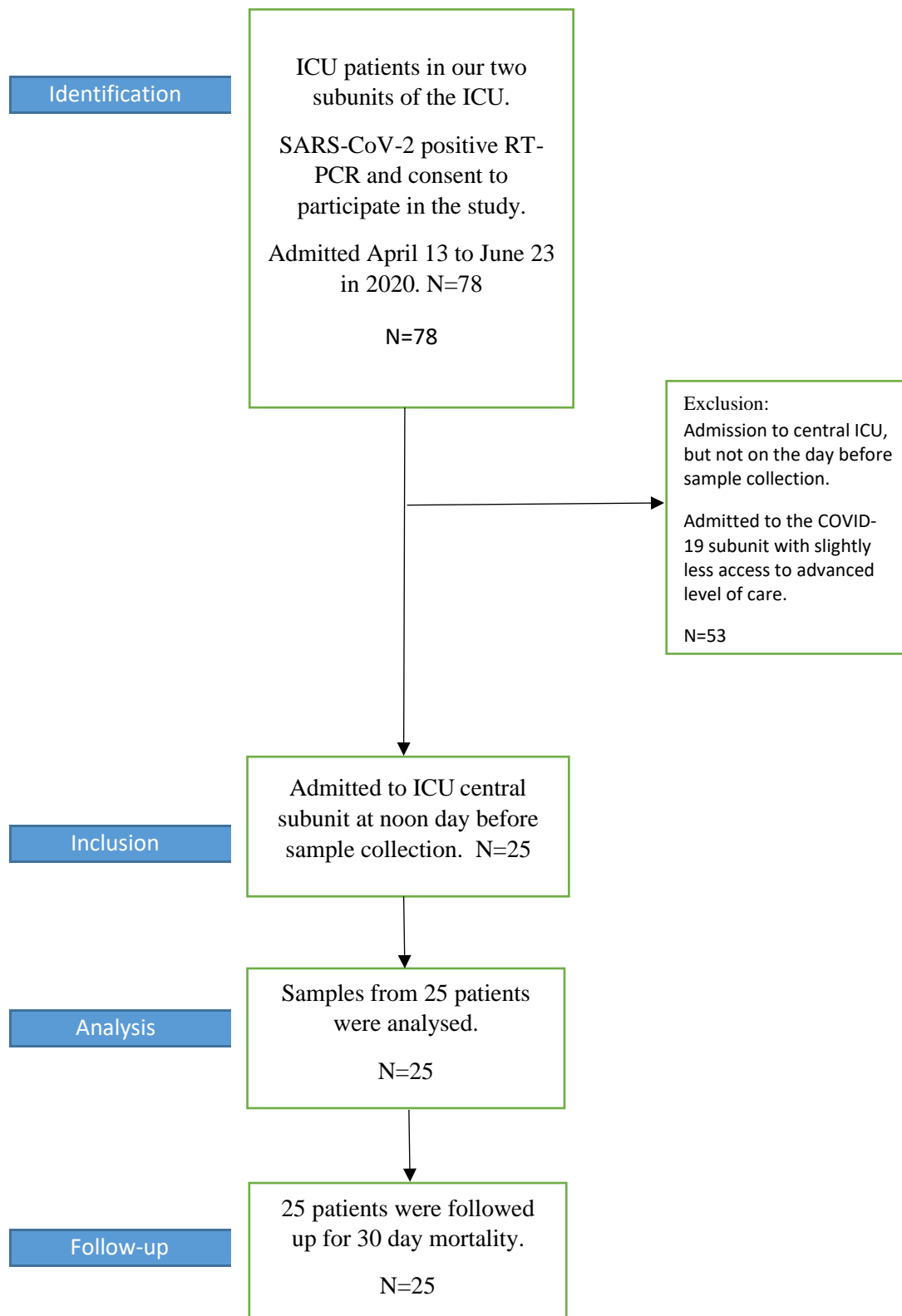

**Figure S1.** Study Flow Diagram.

Supplement: Supplementary file 1 [file biomedicines-10-00934-s001.zip › Figure S1.pdf]
